# Ultra‐Small Air‐Stable Triplet‐Triplet Annihilation Upconversion Nanoparticles for Anti‐Stokes Time‐Resolved Imaging

**DOI:** 10.1002/anie.202308602

**Published:** 2023-09-19

**Authors:** Bolong Zhang, Kieran D. Richards, Beatrice E. Jones, Abigail R. Collins, Rosie Sanders, Sarah R. Needham, Pu Qian, Amoghavarsha Mahadevegowda, Caterina Ducati, Stanley W. Botchway, Rachel C. Evans

**Affiliations:** ^1^ Department of Materials Science and Metallurgy University of Cambridge 27 Charles Babbage Road Cambridge CB3 0FS UK; ^2^ CAS Key Laboratory of Design and Assembly of Functional Nanostructures and Fujian Provincial Key Laboratory of Nanomaterials Fujian Institute of Research on the Structure of Matter Chinese Academy of Sciences Fuzhou Fujian 350002 China; ^3^ Diamond Light Source Didcot Oxfordshire OX11 0QX UK; ^4^ Central Laser Facility Science and Technology Facilities Council Rutherford Appleton Laboratory Harwell Science and Innovation Campus Oxfordshire OX11 0QX UK; ^5^ Materials and Structural Analysis Thermo Fisher Scientific Achtseweg Noord 5 5651 GG Eindhoven The Netherlands; ^6^ The Faraday Institution Quad One Harwell Science and Innovation Campus Didcot UK

**Keywords:** Fluorescence, Lifetime Imaging Microscopy, Nanoparticles, Triplet-Triplet Annihilation, Upconversion

## Abstract

Image contrast is often limited by background autofluorescence in steady‐state bioimaging microscopy. Upconversion bioimaging can overcome this by shifting the emission lifetime and wavelength beyond the autofluorescence window. Here we demonstrate the first example of triplet‐triplet annihilation upconversion (TTA‐UC) based lifetime imaging microscopy. A new class of ultra‐small nanoparticle (NP) probes based on TTA‐UC chromophores encapsulated in an organic–inorganic host has been synthesised. The NPs exhibit bright UC emission (400–500 nm) in aerated aqueous media with a UC lifetime of ≈1 μs, excellent colloidal stability and little cytotoxicity. Proof‐of‐concept demonstration of TTA‐UC lifetime imaging using these NPs shows that the long‐lived anti‐Stokes emission is easily discriminable from typical autofluorescence. Moreover, fluctuations in the UC lifetime can be used to map local oxygen diffusion across the subcellular structure. Our TTA‐UC NPs are highly promising stains for lifetime imaging microscopy, affording excellent image contrast and potential for oxygen mapping that is ripe for further exploitation.

## Graphical abstract

A new class of ultra‐small nanoparticle (NP) probes based on triplet‐triplet annihilation upconversion (TTA‐UC) chromophores in an organic–inorganic ureasil host have been prepared and used to stain living cells as a proof‐of‐concept demonstration of TTA‐UC lifetime imaging for the first time. Fluctuations of the UC lifetime in the stained cells indicate the NPs can be used to map local oxygen diffusion across the subcellular structure.

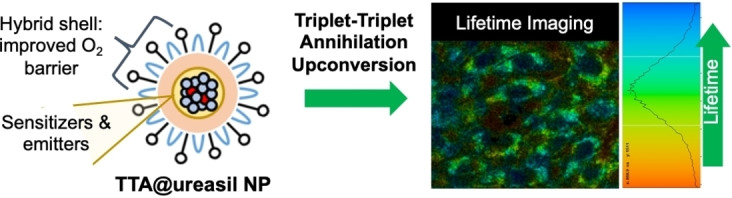

## Introduction

Fluorescence lifetime imaging microscopy (FLIM) is used widely in biomedical imaging to non‐invasively monitor biological processes in real time.[^1^, ^2^] In FLIM, differences in the emission lifetime of fluorophores at each pixel are captured to provide the contrast to produce an image.^[3]^ However, the FLIM contrast may be affected by background autofluorescence from natural biological luminophores, which occurs on the nanosecond timescale.^[1]^ Time‐resolved emission and/or phosphorescence lifetime imaging microscopy (TREM/PLIM) have been demonstrated as an effective solution, in which luminescent probes with much longer relaxation times (hundreds of nanoseconds to microseconds) afford both excellent spatial and temporal discrimination and enable use of time‐gating to discriminate short‐lived autofluorescence.[^4^, ^5^, ^6^] Lanthanide (Ln)[^7^, ^8^, ^9^] and transition metal complexes (e.g. Pt(II),[^4^, ^6^, ^10^, ^11^] Ir(III),[^10^, ^11^, ^12^] Ru(II),[^10^, ^11^, ^12^] Re(I),^[10]^ Pd(II)[^10^, ^11^]) have been successfully used as probes for TREM/PLIM, respectively. However, they are often susceptible to severe oxygen quenching and, consequently, some cytotoxicity related in some part to their extremely long lifetimes which can exceed 100 microseconds.[^11^, ^13^] An attractive alternative are spectral upconversion (UC) probes, which use an anti‐Stokes process to convert two or more photons of low frequency into a single photon of higher frequency.^[14]^ This anti‐Stokes emission, combined with the delayed emission lifetime, allows combined use of both time‐ and spectral‐gating to discriminate from background autofluorescence.^[15]^ To date, there have been limited reports of anti‐Stokes TREM for bioimaging, which primarily use lanthanide(III)‐doped inorganic nanoparticles (NPs) as the luminescent probe.[^15^, ^16^] Ln UCNPs have also been used to sensitise anti‐Stokes emission from Ir(III) complexes, finding application in phototherapy and steady‐state bioimaging.^[17]^ However, all Ln‐based luminophores suffer from low absorption cross‐sections (under direct excitation) and low UC quantum yields due to the associated f‐f‐ electronic transitions. Recently, luminescent NPs based on triplet‐triplet annihilation upconversion (TTA‐UC) have been used for steady‐state spectral‐based bioimaging.[^18^, ^19^, ^20^, ^21^, ^22^] TTA‐UC offers several advantages over Ln UCNP‐sensitised systems for TREM, including relatively high emission quantum yields and a low excitation intensity threshold.[^23^, ^24^] However, to the best of our knowledge, to date TTA‐UC based lifetime imaging in living cells has not yet been reported.

**Figure 1:**
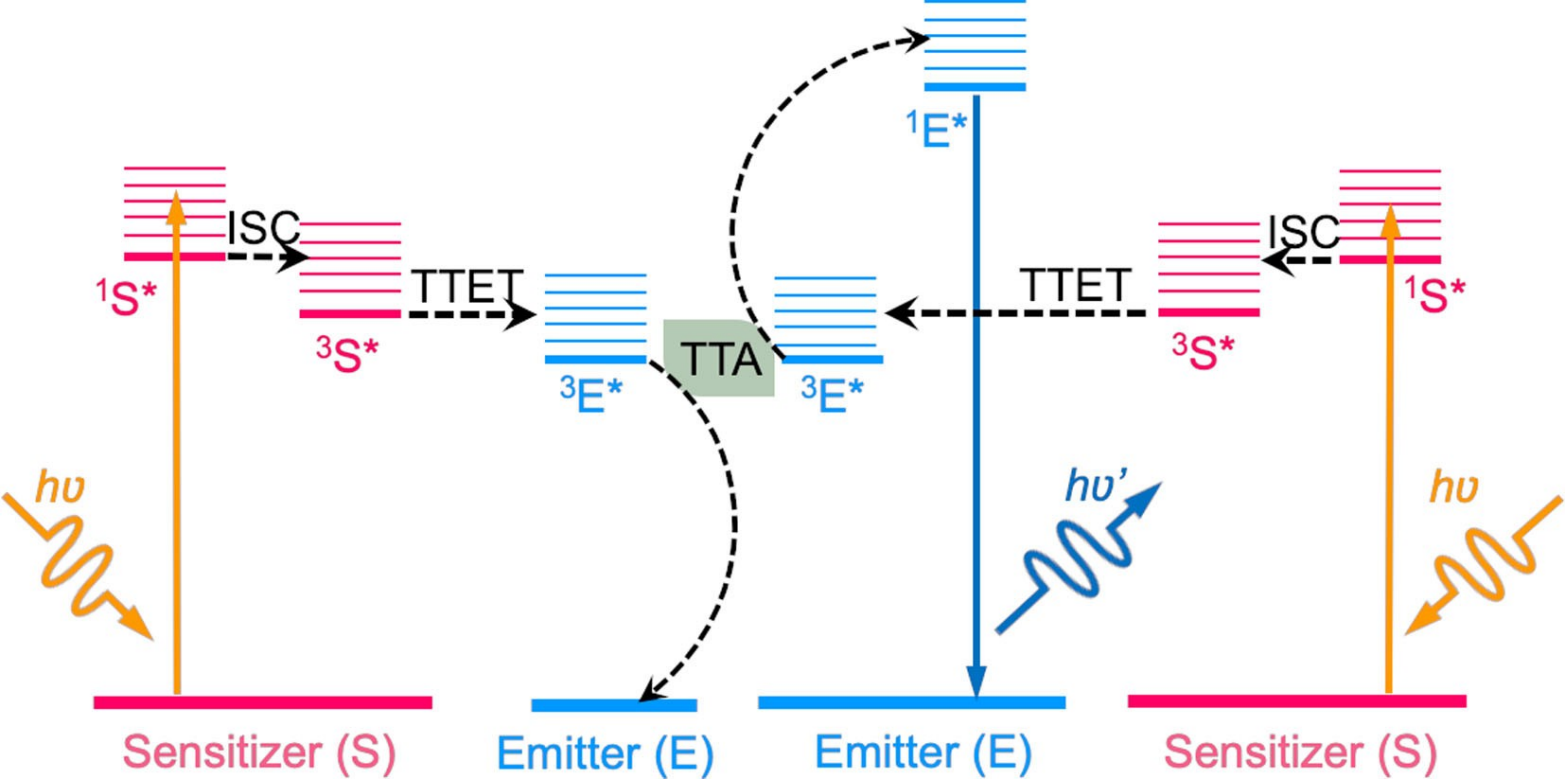
A simplified energy level diagram for the typical TTA‐UC mechanism, where two low‐energy photons (*hv*, orange lines) are absorbed by the sensitizers, transferred to the emitters via triplet‐triplet energy transfer (TTET), and finally converted to one high‐energy photon (*hv*′, blue line) by triplet‐triplet annihilation (TTA).

A typical TTA‐UC system (Figure 1) consists of a luminophore pair of emitters and sensitizers.[^25^, ^26^, ^27^] The sensitizers absorb photons and eventually relax to a triplet excited state via intersystem crossing (ISC). The triplet sensitizers then transfer energy to the emitters via triplet‐triplet energy transfer (TTET), producing triplet emitters. When two triplet emitters collide, their energy is combined, converting one molecule to the singlet excited‐state via the TTA process, which finally relaxes by emitting a single, higher energy photon. TTA‐UC systems have been widely studied in both solution and macroscopic solid‐state materials, for applications such as solar energy harvesting,^[28]^ sensing,[^29^, ^30^, ^31^, ^32^] and light‐emitting devices.^[27]^ However, bioimaging applications require the preparation of the TTA‐UC luminophores as nanoparticles to enable cell uptake.^[19]^ The preparation of TTA‐UC NPs is non‐trivial. Firstly, typical colloidal synthesis procedures normally require the NPs to be homogeneously dispersed in an anti‐solvent, such as water.^[33]^ Thus, to avoid severe aggregation of the hydrophobic organic TTA‐UC chromophores,^[34]^ a suitable amphiphilic host substrate is needed to disperse them. Secondly, long‐term colloidal stability of the NP dispersions is essential for practical use, since on‐going aggregation through Ostwald ripening will alter the photophysical properties. Thirdly, excited triplet states are very effectively quenched by ground‐state O_2_, leading to a reduction of the UC quantum efficiency in air.^[35]^ While oxygen penetration can be prevented by the use of bulk host substrates or lamination in the macroscopic solid‐state,[^32^, ^36^] TTA‐UC chromophores in NPs can be extremely vulnerable to oxygen quenching. To mitigate these challenges, the ideal NP system for TTA‐UC should be dispersible in water, provide long‐term stability, prevent oxygen quenching, and be adaptable to a variety of sensitizer‐emitter pairs.

Herein, we report the first example of the use of TTA‐UC nanoparticles as long‐lived probes for anti‐Stokes TREM on living cells. In previous reports of TTA‐UC for steady‐state spectral imaging, microemulsion synthesis was used to encapsulate the luminophores in water‐stable silica NPs.[^37^, ^38^] Here, we instead use an organic–inorganic hybrid polymer from the ureasil family as an amphiphilic host substrate to prepare TTA‐UC NPs. The ureasil structure consists of poly(alkylene oxide) chains grafted to a siliceous skeleton via urea cross‐linkages.^[39]^ Ureasils combine the biocompatibility of silica,^[40]^ with the advantage of reduced oxygen diffusion rates afforded by the inclusion of the poly(alkylene oxide) chains.^[41]^ Moreover, the chemical and physical properties of ureasils (e.g. hydrophilicity, strength, flexibility) can easily be tuned by varying the poly(alkylene oxide) precursor used.[^42^, ^43^, ^44^] While bulk ureasil materials have been extensively studied for their properties[^45^, ^46^, ^47^, ^48^, ^49^] and applications (e.g. drug delivery,[^50^, ^51^] luminescent solar concentrators,[^52^, ^53^] visible light communications,^[54]^ electrochromic displays),^[55]^ our group has recently reported a precipitation approach for synthesis of fluorescent ureasil NPs (hydrodynamic diameter, *D*_h_≈150 nm) for the first time.^[56]^ The ureasil NPs showed excellent compatibility with embedded organic luminophores, high stability in aqueous dispersions and low biotoxicity. Here, we report a new route to synthesise ultra‐small (≈6 nm) ureasil NPs using emulsion polymerisation, with the goal of improving cell permeability by reducing the particle size. We show that the ureasil NPs readily encapsulate TTA‐UC luminophore pairs. Detailed studies on the photophysical behaviour of the NPs were performed to evaluate the role of the ureasil host on the efficiency and stability of the TTA‐UC emission. Furthermore, we successfully demonstrate the first example of the use of TTA‐UC NPs as probes for TREM to study living cells under ambient physiological conditions.

## Results and Discussion

### Synthesis of TTA‐UC@ureasil NPs

**Figure 2:**
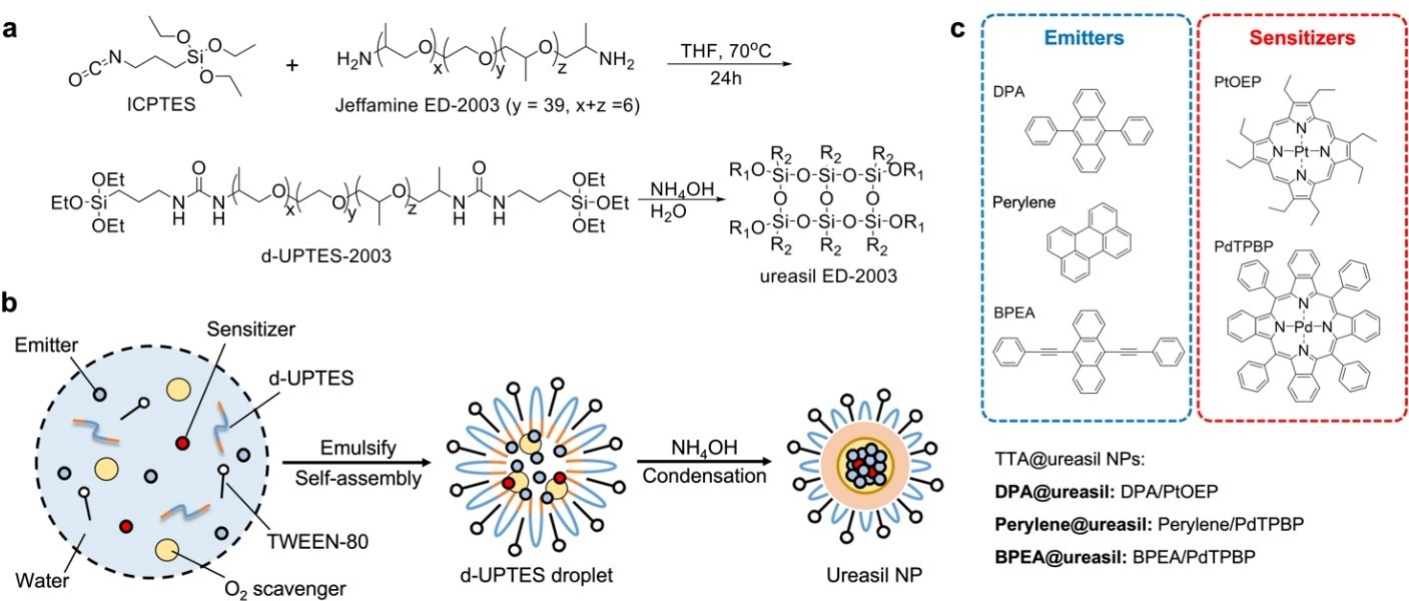
Synthetic route to TTA@ureasil nanoparticles. a. Synthesis of the intermediate d‐UPTES‐ED2003 and the ureasil ED‐2003 (R_1_=−Si−O−Si− or −H, R_2_=−(CH_2_)_3_−NHCONH−Jeffamine). b. Schematic representation of the one‐pot route to TTA@ureasil NPs via emulsification and self‐assembly of the d‐UPTES intermediate and other components, followed by condensation of the ureasil shell. c. Chemical structure of the emitter and sensitizer luminophores and TTA‐UC@ureasil nomenclature.

TTA‐UC NPs were prepared in a two‐step process as illustrated in Figure 2. The body of the NPs is ideally an amphiphilic host that can stabilise the hydrophobic luminophores in an aqueous environment. Jeffamine® ED‐2003 was selected as the host precursor, as its backbone composed of alternating hydrophobic poly(propylene glycol) (PPG) and hydrophilic poly(ethylene glycol) (PEG) blocks (Figure 2a), satisfies this requirement. The ratio and size of the PPG/PEG blocks in the precursor also determines the range of particle sizes that can be accessed.

ED‐2003 was reacted with 3‐(triethoxysilyl)propylisocyanate (ICPTES) in THF to give the diureapropyltriethoxysilane intermediate (d‐UPTES‐ED‐2003). When dispersed in water, d‐UPTES‐ED‐2003 self‐assembles into NPs, with the hydrophilic blocks pointing outwards, and the hydrophobic blocks retained within the core (Figure 2b). The hydrophilic shell helps to disperse the NPs in water, while the hydrophobic core provides good solubility for organic luminophores.^[57]^ Sensitizer‐emitter pairs for TTA‐UC were added to the d‐UPTES‐ED‐2003 aqueous dispersion, along with an oxygen scavenger, bis(methylthiol)methane (BMTM), which was incorporated to enhance the UC efficiency in air.^[58]^ TWEEN‐80® (T80) was also added at this step as an emulsifier to control the final size and shape of the d‐UTPES‐ED‐2003 NPs, and improve the colloidal stability.^[5]^ Upon addition, all hydrophobic components, including the luminophores and BMTM, diffused to the NP core. Finally, NH_4_OH (pH 9) was added to condense the siloxane backbone and form the ureasil shell that encapsulates the inner core, thereby reducing leakage of the contained active material. At pH 9, the particle surface is also expected to be negatively charged (isoelectric point of silica is pH=2), which affords additional electrostatic stabilisation of the colloidal dispersion.

Ureasil NPs were primarily studied using the benchmark TTA‐UC pair of 9,10‐diphenylanthracene (DPA) as the emitter and platinum octaethylporphyrin (PtOEP) as the sensitizer (Figure 2c). Samples are denoted as TTA@ureasil, where TTA is replaced by the emitter molecule used, i.e., DPA@ureasil. The emitter concentration was optimised to the maximum concentration that prevented precipitation from the NP: 1.3 mM in the DPA@ureasil aqueous suspension, and 33 mM with respect to (w.r.t.) the volume of dUPTES‐ED‐2003 used. The sensitizer concentration was then tuned to be the lowest concentration that allowed observation of UC to maximise the UCQY, namely 0.8 μM in aqueous suspension, giving a sensitizer to emitter ratio of 1: 1600. Control samples containing only the sensitizer were also prepared (e.g., PtOEP@ureasil). To demonstrate the versatility of the ureasil NPs as hosts, two additional TTA‐UC systems were also studied, based on *meso*‐tetraphenyl‐tetrabenzoporphine palladium (PdTPBP) as the sensitizer (0.8 μM), and either perylene (perylene@ureasil, 0.32 mM) or 9,10‐bis(phenylethynyl)anthracene (BPEA@ureasil, 1.3 mM) as the emitters. The systems behaved similarly to DPA@ureasil as described in the Supporting Information (Section 3.6 and Figure S6). Full details of the synthetic methods can be found in the Methods section.

**Figure 3:**
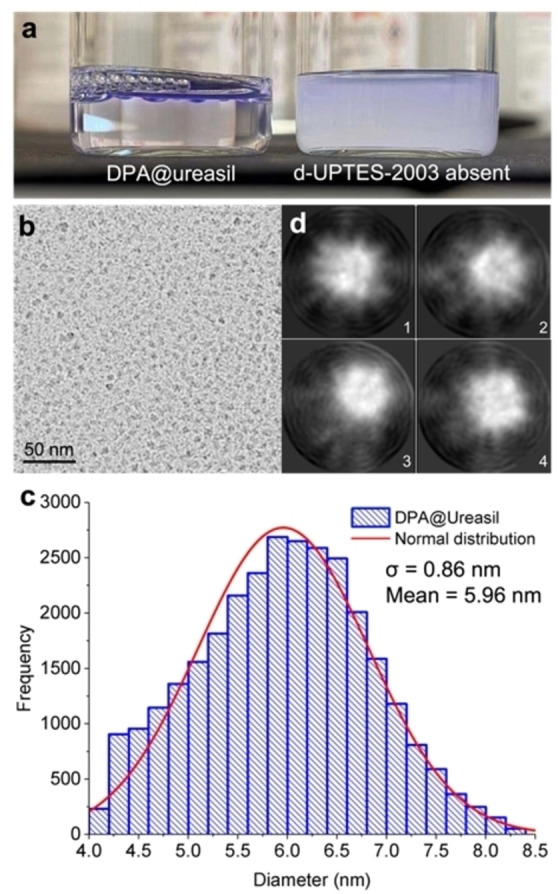
Size and morphology of DPA@ureasil NPs. a. Photograph of suspensions of DPA@ureasil and a counterpart prepared without d‐UPTES‐ED2003 illustrating that NP formation leads to effective solubilisation of the entrapped luminophores. b. Representative cryo‐TEM image of DPA@ureasil and c. Histogram of the corresponding NP diameter and normal distribution fitting analysed over ≈30,000 objects from 171 images. The mean diameter of DPA@ureasil is 5.96±0.01 nm. d. Stacking images of the most populated four NP structures of DPA@ureasil. The spiky features on the particle surface are attributed to luminophore crystallisation upon sample ageing.

Figure 3a shows a photograph comparing the appearance of DPA@ureasil and an analogous sample prepared without d‐UPTES‐2003. The high transparency of DPA@ureasil clearly demonstrates that d‐UPTES‐ED2003 dramatically improves the solubility of the hydrophobic luminophores in water. Dynamic light scattering (DLS) showed that the mean hydrodynamic diameter of the NPs, *D*_h_, is 9.9±0.2 nm with a dispersity index (DI) of 0.088 (see Figure S1, Supporting Information), indicating a high level of uniformity in the size and shape of the NPs, as well as good dispersibility in water. Cryo‐transmission electron microscopy (cryo‐TEM) confirmed the spherical particle shape (see Figure 3b and 3d). The mean diameter of DPA@ureasil from cryo‐TEM is 5.96±0.01 nm with DI of 0.021 (Figure 3c), which is about 4 nm smaller than *D*_h_, as expected. The cryo‐TEM data were further analysed by stacking the images of NPs of similar sizes and shapes. The stacking imaging of the most populated particle structures in DPA@ureasil shows a rugged surface with some spiky features (Figure 3d). These spiky features may be a result of gradual recrystallisation of luminophores from the core due to the elevated concentration.

We note that the DPA@ureasil system has the smallest diameter among reported TTA‐UC NPs.[^18^, ^21^, ^37^] The small size confers significant advantages for bioimaging including: (1) improved penetration depth into living cells; (2) potential for improved imaging resolution and (3) improved long‐term colloidal stability. However, the drawback is that O_2_ can easily diffuse through the shell of DPA@ureasil NPs, despite the O_2_ barrier characteristics of ureasils. We will discuss both the advantages and disadvantages of the ultra‐small size of DPA@ureasil in the following sections.

### Spectroscopic analysis of DPA@ureasil

**Figure 4:**
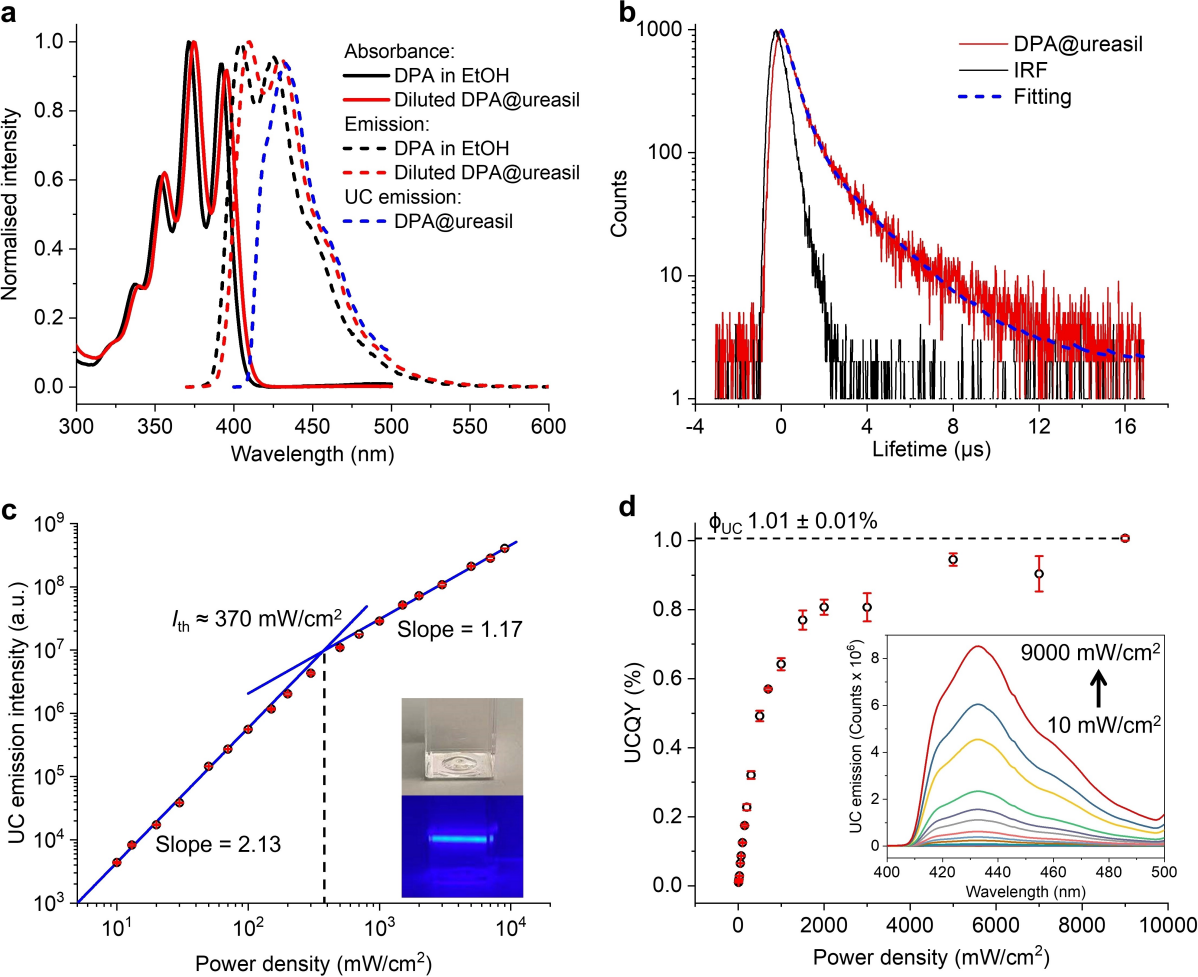
Optical properties of DPA@ureasil. a. Normalised absorption (solid lines) and emission spectra (dashed lines) of DPA in ethanol (1×10^−5^ M, black) and the DPA@ureasil NP suspension (diluted 100‐fold by water, red, *λ*_exc_= 355 nm). The UC emission spectrum (blue) of the parent DPA@ureasil NP suspension (*λ*_exc_=532 nm, short‐pass filter cut off at 500 nm) normalised to the emission spectrum of dilute DPA@ureasil at 432 nm is also shown to reveal the influence of reabsorption on DPA. b. Decay profile (red) and corresponding biexponential fit (blue dashed line) for the UC emission from DPA@ureasil under laser excitation at 532 nm. The instrument response function (IRF) is also shown (black line). c. Double logarithmic plot of UC emission intensity (integrated from 400 to 500 nm) and corresponding linear fits in the low and high excitation regimes. The threshold intensity, *I*_th_, was determined from the intersection of these two regimes (dashed line). Inset: Photographs of DPA@ureasil under ambient light and green (532 nm) laser excitation (500 nm short‐pass filter). The blue UC emission from DPA is clearly visible. d. Upconversion quantum yield of DPA@ureasil and the corresponding UC emission spectrum (inset) with increasing excitation power density. All results in c and d are the mean of three measurements.

The absorption and steady‐state emission properties of DPA@ureasil were investigated next to understand the role of the NP environment. For initial spectroscopic analysis, the native sample was diluted 100‐fold (13 μM DPA in water) to avoid inner filter effects due to the high DPA concentration. We note that dilution should not affect the effective luminophore concentration entrapped within the NP core due to its low water solubility. The absorption profile of the dilute DPA@ureasil NP suspension is identical to that of DPA in ethanol at an equivalent concentration (Figure 4a). This indicates that both the absorbance and scattering of the host ureasil NPs are negligible compared to the absorbance of DPA at this concentration. The fluorescence spectrum of the dilute DPA@ureasil NPs (obtained upon direct excitation of DPA at 355 nm) is also comparable to the DPA ethanolic solution, confirming that the any host emission is negligible.^[56]^ While the absorption and emission spectra of the dilute DPA@ureasil NPs are slightly red‐shifted (≈5 nm) compared to the DPA ethanolic solution, the measured photoluminescence quantum yield (PLQY) is 99±3% for DPA@ureasil, which is comparable to literature reports (97 % in cyclohexane).^[60]^

UC and phosphorescence studies were then performed on the native (non‐dilute) DPA@ureasil NPs. As introduced, the TTA‐UC process is very sensitive to oxygen. While the poly(etheramine) moieties in the ureasil are expected to reduce the oxygen penetration rate,^[41]^ the small diameter of the NPs (≈6 nm) means that this domain is likely to be too thin to completely inhibit O_2_ ingression. UC emission was observed from DPA@ureasil without oxygen scavenger (BMTM), while the intensity is ≈26 times greater in DPA@ureasil with BMTM (Figure S2). Under laser excitation of the sensitizer at 532 nm, an intense blue emission is observed, which is characteristic of UC emission from DPA (Inset of Figure 4c). As shown in Figure 4a, the onset of this UC emission is red‐shifted by ≈25 nm compared to the fluorescence of the dilute sample. This is due to optical reabsorption at this high DPA concentration, as the first vibronic band overlaps strongly with the tail of the absorption band. However, at wavelengths >430 nm, the UC emission profile is the same as that of the dilute sample, indicating that the NP concentration does not affect the optical properties of the embedded luminophores.

The triplet‐triplet energy transfer rate, *φ*_TTET_, of DPA@ureasil is 52 % (Figure S3), which is comparable to similar TTA‐UC NP systems,[^18^, ^20^] but it also means about half of the population of excited sensitisers do not transfer the triplet energy to the emitters. This is likely due to a non‐uniform distribution of luminophores,[^18^, ^61^] which is a trade‐off of the ultra‐small particle size of DPA@ureasil.

The average UC emission lifetime of DPA@ureasil was 0.94 μs (Figure 4b) in air at room temperature, which is more than two orders of magnitude longer than the typical fluorescence lifetime of DPA (7.7 ns in cyclohexane).^[60]^ The long‐lived UC emission is mainly due to indirect excitation of the emissive singlet state via diffusion and collision of the triplet emitters during the TTA process.^[63]^ Two exponential components were required to fit the decay curve (see Figure S4); the shorter‐lived component (τ_1_=0.4 μs, f_1_=71 %) is attributed to the fast decay of the DPA triplet population caused by oxygen quenching, while the longer‐lived component (τ_2_=2.3 μs, f_2_=29 %) is dominated by the rate of diffusion of the DPA triplets in the annihilation step. We note that it was not possible to observe UC emission from the BMTM‐free DPA@ureasil sample due to severe O_2_ quenching of the DPA triplet state. The addition of BMTM significantly decreased the quenching of the UC emission lifetime, which is essential for the TREM study we explore later on.

Figure 4c shows the UC emission intensity of DPA@ureasil, integrated from 400 to 500 nm, as a function of the laser power density. The slope of the linear fit is 2.13 from 10 to 100 mW cm^−2^, and 1.17 from 1000 to 9000 mW cm^−2^, illustrating that DPA@ureasil displays the typical power law dependence observed for TTA‐UC systems.^[62]^ The threshold intensity, *I*_th_, of DPA@ureasil is approximately 370 mW cm^−2^, which is similar to other reported NP systems.[^20^, ^21^] We note there are three factors that affect the *I*_th_ of DPA@ureasil. First, the phosphorescence of PtOEP was not fully quenched. As *φ*_TTET_ is 52 %, about half of the photons absorbed fail to populate the triplet emitters at a given excitation photon density, leading to an increase in *I*_th_. Second, the NP system is not deoxygenated. The residual O_2_ quenching reduces the population of the triplet DPA, which also contributes to the high *I*_th_. Finally, we use a low sensitiser to emitter ratio (1 : 1600), which increases *φ*_TTET_ (and upconversion quantum yield, *φ*_UC_, as discussed below), but also leads to a lower sensitisation rate. This raises the excitation density required to obtain the threshold population of triplet emitters. Since light scattering was insignificant compared to the absorbance of PtOEP in DPA@ureasil at 532 nm (Figure 4a), *φ*_UC_ of DPA@ureasil could be determined by the relative quantum yield measurement approach.^[37]^ The φ_UC_ increases rapidly up to a laser power of 1000 mW cm^−2^, before eventually plateauing to the maximum value, φ_UC_=1.01±0.01 % at 9000 mW cm^−2^ (Figure 4d), which is comparable to other NP systems in air.[^20^, ^21^]

### Stability studies on DPA@ureasil

**Figure 5:**
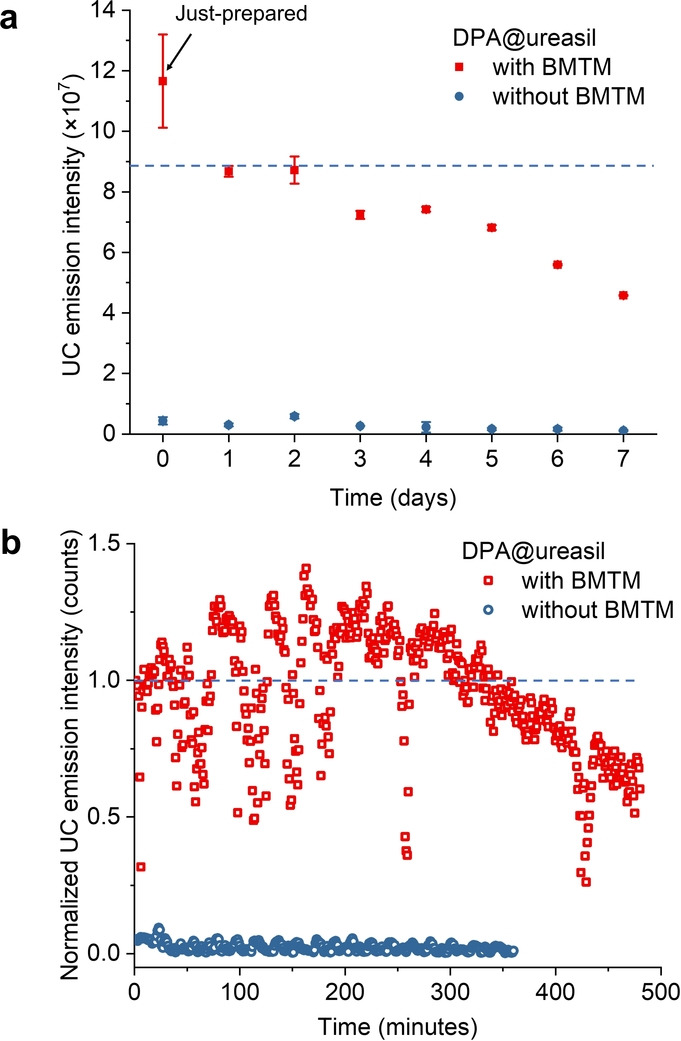
a. Effect of oxygen on the integrated UC emission intensity with time: DPA@ureasil (red squares) and the counterpart sample without BMTM (blue circles) were monitored over eight days (*λ*_exc_=532 nm laser at 2000 mW cm^−2^). All data points are average values over three parallel scans. b. Photostability of DPA@ureasil with and without BMTM under continuous laser excitation (*λ*_exc_=532 nm laser at 2000 mW cm^−2^) as monitored by the normalised UC emission intensity.

The oxygen scavenger significantly improves not only the UC emission intensity, but also the stability of the TTA‐UC NPs. The stability of DPA@ureasil was investigated by monitoring the UC emission intensity daily over one week (Figure 5a). Both samples were monitored and prepared without degassing and were stored under ambient indoor lighting and temperature conditions between measurements, covered by Parafilm^TM^ to avoid solvent evaporation. As expected, the UC emission intensity of DPA@ureasil was significantly higher than the BMTM‐free sample over the entire monitoring period.

Interestingly, the UC emission intensity of DPA@ureasil measured immediately after sample preparation showed some variability, which we attribute to incomplete consumption of residual O_2_ by the scavenger. However, after aging for 24 hours (i.e., Day 1) the error between measurements reduced significantly. As shown in Figure 5, the UC emission intensity of DPA@ureasil (with BMTM) decreased gradually over a week, to 85 % on Day 4 and 53 % on Day 7, relative to the initial intensity. This behaviour is attributed to a combination of DPA aggregation, and photobleaching by the laser in the presence of O_2_. In comparison with the counterpart sample without d‐UPTES‐ED2003 (Figure 3a), in which DPA precipitated within an hour after preparation, the much‐improved shelf life of DPA@ureasil is attributed to the ultra‐small size (6 nm), which improves colloidal stability.

The photostability of DPA@ureasil under ambient conditions was further examined under continuous laser excitation in comparison with the sample without BMTM (Figure 5b). The UC fluorescence of DPA@ureasil gradually increased by ≈25 % of the initial value over 150 minutes irradiation, due to the consumption of residual oxygen, then slowly decreased to ≈60 % over 450 minutes. Nonetheless, we note that the photostability of DPA@ureasil under continuous laser excitation in ambient conditions is significantly longer‐lived than related TTA‐UC NP systems,^[18]^ while many others did not provide data about the long‐term stability.[^20^, ^37^]

### TREM of living cells stained with DPA@ureasil

TTA‐UC materials have shown great potential in steady‐state bioimaging due to their unique anti‐Stokes photoluminescence, low biotoxicity, and high UC quantum yield.[^23^, ^24^] To demonstrate proof‐of‐concept use of the long‐lived UC emission in time‐resolved emission bioimaging, the DPA@ureasil suspension was added to living Chinese hamster ovary (CHO) cells in culture, and TREM analysis performed using a custom confocal microscope (see Supporting Information for further details). To enhance the UC emission, a sample of DPA@ureasil containing a 16‐fold increase in sensitizer concentration was used. The DPA@ureasil suspension was added to the CHO culture media (final concentration of 100 μM) and left in the dark in an incubator (37 °C, 5 % CO_2_, humidified atmosphere) for 1–3 hours to allow the cells to uptake the NPs. The cell staining process is efficient and effortless thanks to the ultra‐small size of DPA@ureasil NPs. No significant changes in the cell morphology were observed 24–48 hours after uptake of DPA@ureasil, suggesting a low toxicity of the NP suspension (see Figure S5). We note that all TREM measurements were performed using freshly prepared NP samples that were diluted in buffer solution to pH 7.

**Figure 6:**
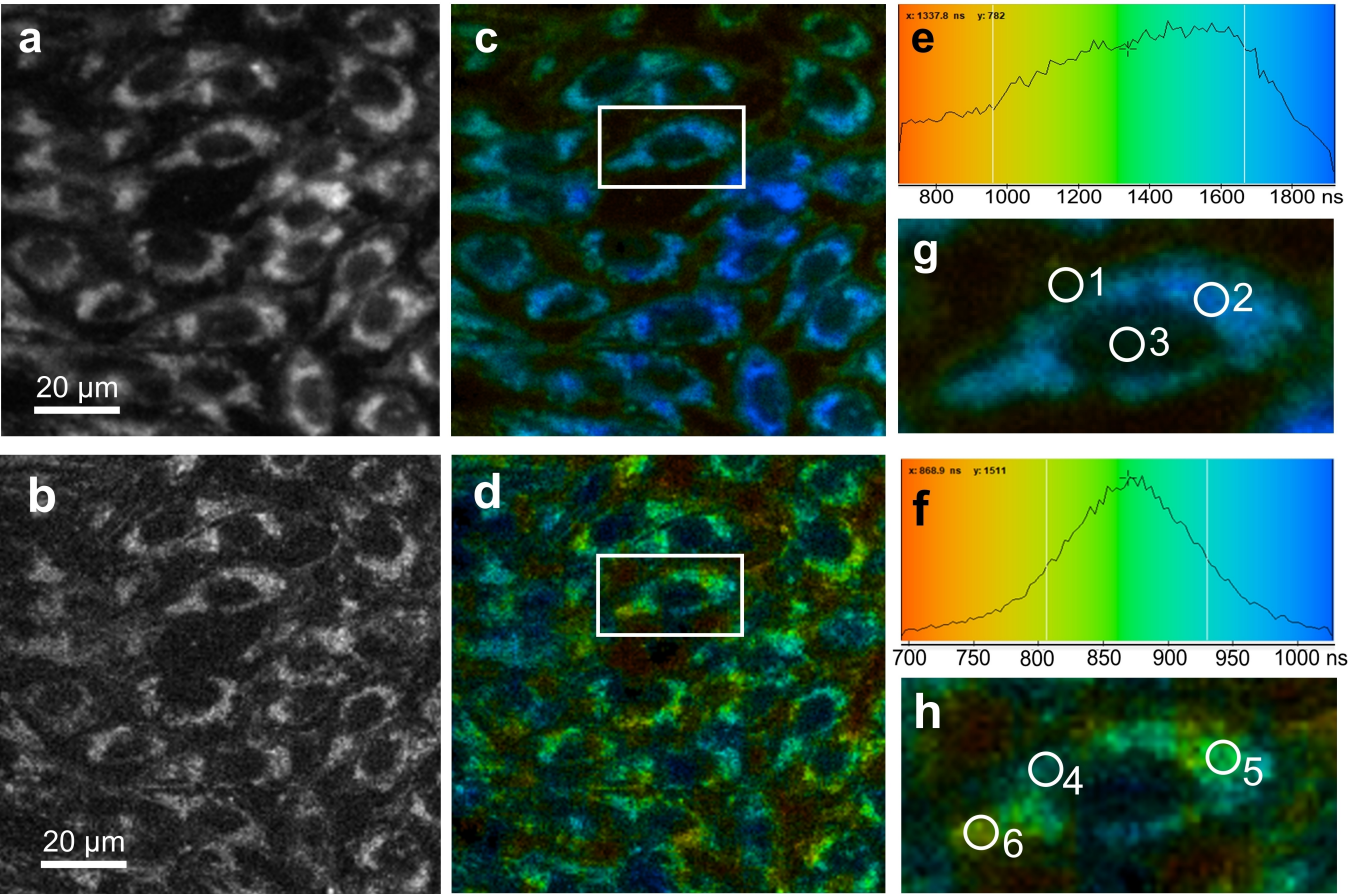
Grey scale images (a. and b.) and TREM images (c. and d.) of CHO cells loaded with DPA@ureasil for 2 hours at 37 °C. Intensity of the emission lifetime distribution in the TREM image (e. and f.). Zoomed images of an individual CHO cell (g. and h. from the white boxes in c and d) and the regions of interest (numbered circles) with different emission decay lifetimes as described in the text. Figures a, c, e, and g were taken under laser excitation (532 nm) of 30 μW, while b, d, f, h were at 19 μW. Field of view: 100 μm. A short pass cut‐off filter (500 nm) was used to allow observation of the anti‐Stokes emission.

The emission signal in Figure 6 is attributed to UC from DPA@ureasil within the CHO cells at 2 hours after staining, taken under two different laser excitation powers (30 μW and 19 μW, 17 and 10 mW cm^−2^, on pulse peak, Figure 6c and d, respectively). The grey scale images show that the DPA@ureasil NPs localise predominantly outside of the nucleus (Figure 6a and b), albeit with some permeation. No obvious aggregation was observed in the images (acquired within 3 hr of staining), indicating DPA@ureasil retains its NP structure. Therefore, the UC lifetimes of the NPs are affected by the local environment in the cell rather than the TTA‐UC luminophores interacting with the cell structures directly.

We note that no signal was observed in control samples without the addition of DPA@ureasil using the 500 nm short‐pass filter. Autofluorescence is also excluded since the main cellular chromophore likely to be excited at 532 nm is flavin adenine dinucleotide, which emits at wavelengths >500 nm on nanosecond timescale (≈5 ns),^[61]^ which is outside of our temporal observation window. This suggests that the long‐lived anti‐Stokes emission of DPA@ureasil is the only source of signal in the TREM analysis, which should in principle be able to enhance contrast of cell imaging.

The average emission lifetimes in CHO cells were 1.5 μs and 0.86 μs under a mean excitation power of 30 μW and 19 μW, respectively (Figures 6e and f). Both lifetimes are similar to the UC lifetime (1 μs) observed in the DPA@ureasil suspension, supporting the observation that DPA@ureasil remains in nanoparticulate form in the cells during the measurement. The excitation dependence of the lifetime is due to the role of O_2_ as a quencher of the TTA‐UC process. A higher excitation intensity leads to a larger population of triplet emitters, while the absolute O_2_ quenching rate remains unchanged. This in turn reduces the relative contribution of the quenching‐related fast‐decay component, leading to a longer emission lifetime. It is worth noting that the increased triplet population may increase the TTA rate in degassed systems. However, here the UC decay profile is dominated by this fast component caused by O_2_ quenching, therefore, a longer lifetime was observed despite the faster TTA rate at higher excitation power.

Further investigation at the sub‐cellular level reveals that the UC lifetime also varies depending on the location within the cell (Figures 6g and h). The O_2_ quenching sensitivity of the ultra‐small DPA@ureasil NPs become a useful tool in mapping the O_2_ diffusion in the cells. At 30 μW excitation, the observed UC lifetime ranges from 0.8–2 μs, depending on the location within the cell (Figure 6g). Emission decays were extracted from three distinct regions to explore this behaviour. The longest lifetime (2.0 μs) is observed in the cytoplasm (region 2), which exceeds the UC lifetime of DPA@ureasil in ambient solution. We attribute this to the probe being present in a lower O_2_ environment, or potentially due to its complexation with an undetermined biomolecule leading to a slower diffusion rate in this cell region compared to solution phase. In the region close to the plasma membrane (region 1), the UC lifetime reduced to ≈1.5 μs, possibly due to faster O_2_ diffusion and access from the outside environment of the cell. In the nucleus (region 3) the UC lifetime is ≈1.7 μs with a much weaker signal intensity. Since the NPs do not stain the nucleus well, we attribute this to a combination of the UC emission signal from both the cytoplasm and the plasma membrane. Although the exact organelle is unknown, we are confident that the subcellular localisation of the probe is neither mitochondria (which generally appear filamentous in CHO cells, unlike the localisation observed here), nor lysosomes and lipid droplets (since staining with Nile red dye did not show co‐localisation). However, the probe localised in the same vicinity as these components that are also found in the general cell cytoplasm.

The UC lifetime distribution (≈0.7 to 1.1 μs) in DPA@ureasil was narrower under lower excitation intensity due to the reduced concentration of triplet states (Figure 6f) and more localized on sub‐cell structures (Figure 6h). We note that the regions of 4, 5, 6 in Figure 6h show the relative variation of the lifetime in the cytoplasm, providing more sub‐cellular detail than in Figure 6g. Similar patterns were also observed in other CHO cells, and the lifetime of some areas in cytoplasm were lower than average (≈880 ns), which suggests that O_2_ diffusion is faster in these areas. These results indicate that our DPA@ureasil NPs may potentially be used to map the O_2_ diffusion rate in living cells, due to the ultra‐small size that affords their uptake through the entire cell structure, combined with their O_2_ sensitivity. We note that fresh NP probe samples were prepared for each set of TREM measurements, which strongly suggests that the observed changes in UC lifetime are primarily associated with the local O_2_ environment in the cell, rather than sample ageing. This is an exciting observation since O_2_ diffusion mapping can be a useful tool to examine disease or damages in living cells, such as the hypoxia environment in tumour cells.^[64]^

## Conclusion

In summary, water‐dispersible ureasil NPs with ultra‐small diameter (≈6 nm) have been successfully synthesised by emulsion polymerisation and exhibit TTA‐UC activity in aerated aqueous media at room temperature. Addition of the oxygen scavenger BMTM greatly enhanced the UC emission intensity, leading to a maximum UC quantum yield of ≈1 %, threshold intensity of 370 mW cm^−2^ and UC lifetime of 0.94 μs for the model DPA@ureasil system. It is worth noting that we have not attempted to optimise the sensitier/emitter ratio for this system and a higher UC quantum yield may be achievable. The DPA@ureasil dispersion showed reasonable long‐term stability in air, retaining ≈50 % of its initial UC emission intensity after 7 days. We attribute this to the combined activity of the ultra‐small NP size coupled with the oxygen scavenger and the improved barrier properties of the organic–inorganic ureasil compared to pure SiO_2_ NPs. DPA@ureasil NPs were successfully used to label live CHO cells and exhibited low cytotoxicity after 24 hours. The long‐lived anti‐Stokes UC emission could be easily distinguished from autofluorescence using TREM, leading to improved image contrast. Interestingly, fluctuations in the emission lifetime were observed at the sub‐cellular level, suggesting that DPA@ureasil NPs could find application as local environment probes, in particular oxygen mapping. Future studies will explore the effect of excitation power density at higher resolution to provide further insight into the sensitivity of these probes to O_2_ concentration, and other key parameters such as pH, temperature, and viscosity at the sub‐cellular level, with the aim of to fully unlocking the potential of TTA‐UC for TREM imaging.

## Data Availability

All data are available from the corresponding author upon reasonable request.

## Author Contributions

B.Z and R.C.E. developed the concept. B.Z. developed the synthetic route to the NPs and performed all spectroscopy measurements and analysis. KDR analysed the TEM data. BEJ and ARC prepared NP samples for TREM. RS and SRN performed cell viability studies. PQ and AM performed TEM measurements under the supervision of CD. SWB performed TREM measurements on cells. B.Z. and R.C.E. drafted the manuscript in consultation with all authors. R.C.E. acquired funding and directed the project.

## Conflict of interest

The authors declare no conflict of interest.

1

## Supporting information

As a service to our authors and readers, this journal provides supporting information supplied by the authors. Such materials are peer reviewed and may be re‐organized for online delivery, but are not copy‐edited or typeset. Technical support issues arising from supporting information (other than missing files) should be addressed to the authors.

Supporting Information

## Data Availability

The data that support the findings of this study are available from the corresponding author upon reasonable request.

## References

[1] R. Datta , T. M. Heaster , J. T. Sharick , A. A. Gillette , M. C. Skala , J. Biomed. Opt. 2020, 25, 071203. 32406215 10.1117/1.JBO.25.7.071203 PMC7219965

[2] M. Y. Berezin , S. Achilefu , Chem. Rev. 2010, 110, 2641–2684. 20356094 10.1021/cr900343z PMC2924670

[3] K. Suhling , P. M. W. French , D. Phillips , Photochem. Photobiol. Sci. 2005, 4, 13–22. 15616687 10.1039/b412924p

[4] S. W. Botchway , M. Charnley , J. W. Haycock , A. W. Parker , D. L. Rochester , J. A. Weinstein , J. A. G. Williams , Proc. Natl. Acad. Sci. USA 2008, 105, 16071–16076. 18852476 10.1073/pnas.0804071105 PMC2570970

[5] S. S. Howard , A. Straub , N. G. Horton , D. Kobat , C. Xu , Nat. Photonics 2013, 7, 33–37. 23472061 10.1038/nphoton.2012.307 PMC3587172

[6] E. Baggaley , S. W. Botchway , J. W. Haycock , H. Morris , I. V. Sazanovich , J. A. G. Williams , J. A. Weinstein , Chem. Sci. 2014, 5, 879–886.

[7] A. Beeby , S. W. Botchway , I. M. Clarkson , S. Faulkner , A. W. Parker , D. Parker , J. A. G. Williams , J. Photochem. Photobiol. B 2000, 57, 83–89. 11154087 10.1016/s1011-1344(00)00070-1

[8] C. P. Montgomery , B. S. Murray , E. J. New , R. Pal , D. Parker , Acc. Chem. Res. 2009, 42, 925–937. 19191558 10.1021/ar800174z

[9] X. Zhu , X. Wang , H. Zhang , F. Zhang , Angew. Chem. Int. Ed. 2022, 61, e202209378. 10.1002/anie.202209378 35918764

[10] E. Baggaley , J. A. Weinstein , J. A. G. Williams , Coord. Chem. Rev. 2012, 256, 1762–1785.

[11] T. Yoshihara , Y. Hirakawa , M. Hosaka , M. Nangaku , S. Tobita , J. Photochem. Photobiol. C 2017, 30, 71–95.

[12] J. Zhou , J. Li , K. Y. Zhang , S. Liu , Q. Zhao , Coord. Chem. Rev. 2022, 453, 214334.

[13] M. Ethirajan , Y. Chen , P. Joshi , R. K. Pandey , Chem. Soc. Rev. 2011, 40, 340–362. 20694259 10.1039/b915149b

[14] H. Chen , B. Ding , P. Ma , J. Lin , Adv. Drug Delivery Rev. 2022, 188, 114414. 10.1016/j.addr.2022.114414 35809867

[15] H. Li , M. Tan , X. Wang , F. Li , Y. Zhang , L. Zhao , C. Yang , G. Chen , J. Am. Chem. Soc. 2020, 142, 2023–2030. 31910008 10.1021/jacs.9b11641

[16] X. Liu , A. Skripka , Y. Lai , C. Jiang , J. Liu , F. Vetrone , J. Liang , Nat. Commun. 2021, 12, 6401. 34737314 10.1038/s41467-021-26701-1 PMC8568918

[17a] W. Lv , T. Yang , Q. Yu , Q. Zhao , K. Y. Zhang , H. Liang , S Liu , F. Li , W. Huang , Adv. Sci. 2015, 2, 1500107; 10.1002/advs.201500107 PMC5115315 27980906

[17b] J. Zhao , S. Sun , X. Li , W. Zhang , S. Gou , ACS Appl. Bio Mater. 2020, 3, 252. 10.1021/acsabm.9b00774 35019441

[18] S. Mattiello , A. Monguzzi , J. Pedrini , M. Sassi , C. Villa , Y. Torrente , R. Marotta , F. Meinardi , L. Beverina , Adv. Funct. Mater. 2016, 26, 8447–8454.

[19] L. Huang , E. Kakadiaris , T. Vaneckova , K. Huang , M. Vaculovicova , G. Han , Biomaterials 2019, 201, 77–86. 30802685 10.1016/j.biomaterials.2019.02.008 PMC6467534

[20] L. Huang , T. Le , K. Huang , G. Han , Nat. Commun. 2021, 12, 1898. 33772017 10.1038/s41467-021-22282-1 PMC7997900

[21] D. C. Thévenaz , A. Monguzzi , D. Vanhecke , R. Vadrucci , F. Meinardi , Y. C. Simon , C. Weder , Mater. Horiz. 2016, 3, 602–607.

[22] L. Zeng , L. Huang , J. Han , G. Han , Acc. Chem. Res. 2022, 55, 2604–2615. 36074952 10.1021/acs.accounts.2c00307

[23] Y. C. Simon , C. Weder , J. Mater. Chem. 2012, 22, 20817–20830.

[24] Y. Zhou , F. N. Castellano , T. W. Schmidt , K. Hanson , ACS Energy Lett. 2020, 5, 2322–2326.

[25] J. Alves , J. Feng , L. Nienhaus , T. W. Schmidt , J. Mater. Chem. C 2022, 10, 7783–7798.

[26] S. E. Seo , H.-S. Choe , H. Cho , H. Kim , J.-H. Kim , O. S. Kwon , J. Mater. Chem. C 2022, 10, 4483–4496.

[27] C. Gao , W. W. H. Wong , Z. Qin , S. Lo , E. B. Namdas , H. Dong , W. Hu , Adv. Mater. 2021, 33, 2100704. 10.1002/adma.202100704 34596295

[28] A. Monguzzi , R. Tubino , S. Hoseinkhani , M. Campione , F. Meinardi , Phys. Chem. Chem. Phys. 2012, 14, 4322–4332. 22370856 10.1039/c2cp23900k

[29] S. M. Borisov , C. Larndorfer , I. Klimant , Adv. Funct. Mater. 2012, 22, 4360–4368.

[30] M. P. Jewell , M. D. Greer , A. L. Dailey , K. J. Cash , ACS Sens. 2020, 5, 474–480. 31912733 10.1021/acssensors.9b02252

[31] D. Yildiz , C. Baumann , A. Mikosch , A. J. C. Kuehne , A. Herrmann , R. Göstl , Angew. Chem. Int. Ed. 2019, 58, 12919–12923. 10.1002/anie.201907436 PMC6772058 31265744

[32] A. L. Hagstrom , H.-L. Lee , M.-S. Lee , H.-S. Choe , J. Jung , B.-G. Park , W.-S. Han , J.-S. Ko , J.-H. Kim , J.-H. Kim , ACS Appl. Mater. Interfaces 2018, 10, 8985–8992. 29441781 10.1021/acsami.7b17789

[33] S. C. Thickett , R. G. Gilbert , Polymer 2007, 48, 6965–6991.

[34] J. Zhao , S. Ji , H. Guo , RSC Adv. 2011, 1, 937–950.

[35] S. H. C. Askes , V. C. Leeuwenburgh , W. Pomp , H. Arjmandi-Tash , S. Tanase , T. Schmidt , S. Bonnet , ACS Biomater. Sci. Eng. 2017, 3, 322–334. 28317022 10.1021/acsbiomaterials.6b00678 PMC5350605

[36] R. R. Islangulov , J. Lott , C. Weder , F. N. Castellano , J. Am. Chem. Soc. 2007, 129, 12652–12653. 17900120 10.1021/ja075014k

[37] Q. Liu , T. Yang , W. Feng , F. Li , J. Am. Chem. Soc. 2012, 134, 5390–5397. 22369318 10.1021/ja3003638

[38] O. S. Kwon , H. S. Song , J. Conde , H. Kim , N. Artzi , J.-H. Kim , ACS Nano 2016, 10, 1512–1521. 26727423 10.1021/acsnano.5b07075

[39] V. de Zea Bermudez , L. D. Carlos , L. Alcácer , Chem. Mater. 1999, 11, 569–580.

[40] Y. Huang , P. Li , R. Zhao , L. Zhao , J. Liu , S. Peng , X. Fu , X. Wang , R. Luo , R. Wang , Z. Zhang , Biomed. Pharmacother. 2022, 151, 113053. 35594717 10.1016/j.biopha.2022.113053

[41] M. Minelli , M. G. De Angelis , F. Doghieri , M. Marini , M. Toselli , F. Pilati , Eur. Polym. J. 2008, 44, 2581–2588.

[42] I. Meazzini , N. Willis-Fox , C. Blayo , J. Arlt , S. Clément , R. C. Evans , J. Mater. Chem. C 2016, 4, 4049–4059.

[43] A. Collins , T. Southern , G. Lyu , M. J. Bennison , R. C. Evans , Proc. SPIE 11367, Photosensitive Materials and their Applications 2020, 113670Y, 10.1117/12.2564510.

[44] E. F. Molina , C. R. N. Jesus , L. A. Chiavacci , S. H. Pulcinelli , V. Briois , C. V. Santilli , J. Sol-Gel Sci. Technol. 2014, 70, 317–328.

[45] L. D. Carlos , R. A. Sá Ferreira , R. N. Pereira , M. Assunção , V. de Zea Bermudez , J. Phys. Chem. B 2004, 108, 14924–14932.

[46] A. L. A. Moura , L. K. de Oliveira , K. J. Ciuffi , E. F. Molina , J. Mater. Chem. A 2015, 3, 16020–16032.

[47] M. Paredes Zaldivar , C. V. Santilli , C. A. Peniche Covas , S. H. Pulcinelli , J. Therm. Anal. Calorim. 2017, 130, 791–798.

[48] G. Palácio , S. H. Pulcinelli , R. Mahiou , D. Boyer , G. Chadeyron , C. V. Santilli , ACS Appl. Mater. Interfaces 2018, 10, 37364–37373. 30346685 10.1021/acsami.8b11149

[49] G. Lyu , T. J. F. Southern , B. L. Charles , M. Roger , P. Gerbier , S. Clément , R. C. Evans , J. Mater. Chem. C 2021, 9, 13914–13925. 10.1039/d1tc02794h PMC8515938 34745631

[50] C. V. Santilli , L. A. Chiavacci , L. Lopes , S. H. Pulcinelli , A. G. Oliveira , Chem. Mater. 2009, 21, 463–467.

[51] B. B. Caravieri , N. A. M. de Jesus , L. K. de Oliveira , M. D. Araujo , G. P. Andrade , E. F. Molina , ACS Appl. Bio Mater. 2019, 2, 1875–1883. 10.1021/acsabm.8b00798 35030677

[52] M. M. Nolasco , P. M. Vaz , V. T. Freitas , P. P. Lima , P. S. André , R. A. S. Ferreira , P. D. Vaz , P. Ribeiro-Claro , L. D. Carlos , J. Mater. Chem. A 2013, 1, 7339–7350.

[53] A. Kaniyoor , B. Mckenna , S. Comby , R. C. Evans , Adv. Opt. Mater. 2016, 4, 444–456.

[54] A. R. Bastos , G. Lyu , T. Silvério , P. S. André , R. C. Evans , R. A. S. Ferreira , Cell Rep. Phys. Sci. 2020, 1, 100041.

[55] P. Barbosa , L. Rodrigues , M. Silva , M. Smith , A. Gonçalves , E. Fortunato , J. Mater. Chem. 2010, 20, 723–730.

[56] I. Meazzini , S. Comby , K. D. Richards , A. M. Withers , F. X. Turquet , J. E. Houston , R. M. Owens , R. C. Evans , J. Mater. Chem. B 2020, 8, 4908–4916. 32315019 10.1039/d0tb00100g

[57] P. Duan , N. Yanai , H. Nagatomi , N. Kimizuka , J. Am. Chem. Soc. 2015, 137, 1887–1894. 25599418 10.1021/ja511061h

[58] D. Dzebo , K. Moth-Poulsen , B. Albinsson , Photochem. Photobiol. Sci. 2017, 16, 1327–1334. 28726960 10.1039/c7pp00201g

[59] T. Yu , Y. Liu , Y. Zeng , J. Chen , G. Yang , Y. Li , Chem. Eur. J. 2019, 25, 16270–16276. 31587399 10.1002/chem.201904025

[60] Y. Fujiwara , R. Ozawa , D. Onuma , K. Suzuki , K. Yoza , K. Kobayashi , J. Org. Chem. 2013, 78, 2206–2212. 23323674 10.1021/jo302621k

[61] C. Gao , B. Zhang , C. R. Hall , L. Li , Y. Chen , Y. Zeng , T. A. Smith , W. W. H. Wong , Phys. Chem. Chem. Phys. 2020, 22, 6300–6307. 32133470 10.1039/c9cp06311k

[62] A. Monguzzi , J. Mezyk , F. Scotognella , R. Tubino , F. Meinardi , Phys. Rev. B 2008, 78, 195112.

[63] N. Yanai , N. Kimizuka , Chem. Commun. 2016, 52, 5354–5370. 10.1039/c6cc00089d 26947379

[64] A. Raza , H. E. Colley , E. Baggaley , I. V. Sazanovich , N. H. Green , J. A. Weinstein , S. W. Botchway , S. MacNeil , J. W. Haycock , Sci. Rep. 2017, 7, 10743. 28878302 10.1038/s41598-017-11153-9 PMC5587740

