# Supplementary material for: Ultra‐Small Air‐Stable Triplet‐Triplet Annihilation Upconversion Nanoparticles for Anti‐Stokes Time‐Resolved Imaging
Source: Angew Chem Int Ed Engl. 2023 Sep 19;62(47):e202308602. doi: 10.1002/anie.202308602 (PMC10952532; doi:10.1002/anie.202308602)
Supplement: Supplementary file 1 — Supporting Information [file ANIE-62-0-s001.pdf]

Angewandte Chemie
Eine Zeitschrift der Gesellschaft Deutscher Chemiker

Supporting Information

Ultra-Small Air-Stable Triplet-Triplet Annihilation Upconversion Nanoparticles for Anti-Stokes Time-Resolved Imaging

*B. Zhang, K. D. Richards, B. E. Jones, A. R. Collins, R. Sanders, S. R. Needham, P. Qian, A. Mahadevegowda, C. Ducati, S. W. Botchway, R. C. Evans**

TABLE OF CONTENTS

1 Materials **3**

2 Characterisation **4**

2.1 Cryo-EM data collection and processing *4*

2.2 Spectroscopic Analysis *5*

2.3 Lifetime imaging of CHO cells using DPA@ureasil *6*

3 Supporting Experimental Data **8**

3.1 Dynamic Light Scattering (DLS) *8*

3.2 Effect of Oxygen on Upconversion Emission for TTA@Ureasils *9*

3.3 The TTET rate of DPA@ureasil. *10*

3.4 UC emission lifetime decay studies *11*

3.5 Cell imaging studies *12*

3.6 Other TTA-UC@ureasil systems *12*

4 References **14**

1 Materials

1.1 General information

Jeffamine® ED-2003, 3-(triethoxy-silyl)propyl isocyanate (ICPTES, 95%), 9,10-diphenylanthracene (DPA, 97%), platinum(II) 2,3,7,8,12,13,17,18-octaethyl-21H,23H-porphyrin (PtOEP, 97%), perylene (99%), 9,10-bis(phenylethynyl)anthracene (BPEA, 97%), bis(methylthio)methane (BMTM, 99%), TWEEN® 80 (T-80) and ammonium hydroxide (NH_4OH, 35% w/w aqueous solution) were purchased from Sigma-Aldrich and used as received. *meso*-Tetraphenyl-tetrabenzoporphine palladium (PdTPBP) was purchased from Porphyrin Products and used as received. All organic solvents were used as received without further purification. Water was obtained from a Millipore Simak 2 water purification system.

1.2 Preparation of TTA-UC@ureasil nanoparticles

Jeffamine ED-2003 (200 g, 105 mmol) was mixed with ICPTES (52 g, 210 mmol) in THF (250 mL) and stirred for 24 h at 70°C under N_2. The resulting solution was stored in sealed glass vials for later use without further purification.

NP dispersions containing different sensitiser/emitter pairs were prepared as follows:

DPA@ureasil: The d-UPTES-ED2003 stock solution (400 µL), T80 (200 mg), DPA solution (10 mM in THF, 400 µL) and PtOEP solution (0.5 mM in THF, 5 µL) were mixed by gently shaking in a glass vial. The solvent was then removed by rota-evaporation at 40°C for 30 min. Then, BMTM (40 µL) was added to the mixture and stirred for 2 min. NH_4OH aqueous solution (1.2% w/w, 3 mL) was then added to the vial and sonicated for 2 min. The solution was incubated for 1 h and filtered with a 0.2 µm nylon syringe filter, giving a clear TTA@ureasil NP dispersion with a faint pink colour. The concentration of DPA was 1.3 mM in the DPA@ureasil aqueous suspension, 33 mM to the volume of dUPTES-ED-2003 used. The concentration of PtOEP was 0.8 µM in aqueous suspension, giving sensitiser to emitter ratio 1: 1600. A control sample (**PtOEP@ureasil**) containing just the PtOEP sensitiser (0.8 µM) and no emitter was also prepared.

BPEA@ureasil and **Perylene@ureasil** were prepared following the same procedure, with substitution of the sensitiser to *meso*-tetraphenyl-tetrabenzoporphine palladium (PdTPBP) (0.8 µM), and either perylene (0.32 mM) or 9,10-bis(phenylethynyl)anthracene (BPEA) (1.3 mM) as the emitters.

2 Characterisation

2.1 Cryo-EM data collection and processing

The original DPA@ureasil suspension was diluted 2-fold by water and filtered by a 0.2 µm nylon syringe filter before the cryo-TEM measurement. 3 µL suspension liquid was applied on a glow-discharged QuantiFiol R1.2/1.3 300 mesh Cu EM grid. The grid was plunge-frozen into liquid ethane using a FEI Vitrobot Mark IV, in which its sample chamber was set to 100% humidity at 4 °C. Grid was stored in liquid nitrogen until use. Cryo-EM data were collected on a Thermo Fisher Titan Krios G3i cryogenic electron microscope equipped with a Falcon 4 direct electron detector at the Cambridge Pharmaceutical Cryo-EM Consortium.[1] The microscope was operated at 300 kV with a nominal magnification of 120,000 x. At specimen level, this magnification corresponds to 0.65 Å/pixel. Counting mode was selected on camera for data collection. A total dose of 48.31 electrons per $Å^2$ within 12.21 seconds exposure time was fractioned into 42 frames, resulting in an electron fluence of 1.15 $e^-/Å^2$/frame. Movie collection was controlled using EPU 2.8 (ThermoFisher Scientific) with one exposure per hole in aberration-free image shift mode (AFIS). In total, 209 movies were collected from a single grid with a defocus of -4.0 µm.

All movie stacks were motion corrected within RELION 3.1[2] on a 5 x 5 patches. The motion-corrected images were CTF (contrast transfer function) corrected using CTFFIND.[3] Particle coordinates were obtained using cisTEM[4] initially and were transferred to RELION for particle picking. In total, 66788 particles were picked up from good images after rejection of all bad images, such as empty, mis-hit, drifted and ice contaminated. A particle box size of 230 pixels, corresponding 149.5 Å (0.65 Å per pixel length), which well-covered a single particle was selected. Reference-free two-dimension (2D) classification in 50 classes was performance in RELION, 62.4% particle entered the first 9 classes based on class distribution statistical analysis. Four of them are shown in Figure 3d in the main manuscript.

ilastik® was used to segment the raw cryo-TEM data.[5] This was performed using pixel classification using all features selected at all length scales. The classifier was trained manually on at least (10) images per dataset using two labels. All pixels of all images in the dataset are assigned a value of 1 or 2 based on the most probable label. This produces simple segmentation images, which were then binned by x4 prior to segmentation to reduce processing times. ImageJ® was used to prepare the segmented images for analysis.[6] This was done by binarizing the image such that the nanoparticle pixels had a value of 255 while

the background pixels had a value of 0. The ‘analyze particles’ tool was used to convert the binarised images into a series of ellipses with x, y coordinates, and radii. The fitting was constrained to particles within a size of 200-800 pixels and a circularity parameter of 0.60 - 1.00.

2.2 Spectroscopic Analysis

Samples were transferred to sealed cuvette (10 mm pathlength) to avoid solvent evaporation during all measurements unless otherwise specified. The transmittance of all filters used in this paper was read by a DS5 spectrometer (Edinburgh Instruments Ltd.), taking the average over 10 parallel measurements at the wavelength used.

UV-Vis absorption spectra of all samples were recorded with a DS5 spectrometer (Edinburgh Instruments Ltd.). Baseline correction was applied for all spectra unless otherwise specified. Fluorescence emission spectra were recorded on a Fluorolog-3 spectrophotometer (Horiba Jobin Yvon) and corrected for the wavelength response of the system. The excitation and emission slits were adjusted so that the maximum PL intensity was within the range of linear response of the detector.

UC emission spectra, phosphorescence spectra, I_{th} and UCQY measurements were performed with an FLS 1000 spectrometer (Edinburgh Instruments Ltd.) equipped with a Visible PMT-980 detector. All measurements were performed at room temperature without any deoxygenation process. DPA@ureasil samples were excited with a 532 nm laser (MGL-III-532, 200 mW) and a short-pass filter (cut-off 500 nm, Thorlabs) placed in front of the detector, while the perylene@ureasil and BPEA@ureasil samples were excited with a 635 nm laser (EPL-635, 5 mW) and a short-pass filter (cut-off 600 nm, Thorlabs).

Before each measurement, the 532 nm laser was carefully tuned to the desired power by a laser power meter (Thorlabs). The sample cuvette was scanned from 400 nm to 500 nm in a gradient of 1 nm under 0.1 second collection time. The slit width of the detector is 1 nm. The collected spectra were corrected by the transmittance of the filters used.

The UCQY was measured via the relative QY approach using Lumogen F Red 305 (BASF, 1×10^{-5} M in $CHCl_3$, PLQY = 96%[7]) was used as the reference luminophore. The UCQY was determined by the following equation:

$$UCQY = PLQY_R \times \frac{E_S}{E_R} \times \frac{(1-0.1^{A_R})}{(1-0.1^{A_S})} \times (\frac{n_S}{n_R})^2 \times \frac{P_R}{P_S}$$

where PLQY, *E, A, n, P* stand for the photoluminescence quantum yield, the integrated emission intensity, the absorbance, the refractive index, and the laser power, respectively, while the *S* and *R* subscripts refer to sample and the reference.

UC decay measurements were performed on the FLS1000 spectrometer using the multi-channel scaling (MCS) single photon counting technique to afford microsecond resolution with a PMT-980 detector. A short-pass filter (cut-off 500 nm, Thorlabs) was placed in front of the detector. Excitation was performed with MGL-III-532 laser. The pulse repetition rate was 50 kHz (20 μs), the laser power was set to its maximum, and the pulse width was gradually increased until more than 1000 counts/s was observed at the detection wavelength (450 nm). The instrument response function (IRF) was measured using Ludox™ solution using a neutral density filter (OD=3) and without the short-pass filter. Data-fitting were performed as individual fits to each emission decay using a multiexponential decay function using the FAST software package (Edinburgh Instruments). The goodness of fit was assessed using the reduced chi-square statistics, χ^2, and the randomness of the residuals.

2.3 Lifetime imaging of CHO cells using DPA@ureasil

All cell culture reagents, unless otherwise stated, were purchased from Thermofisher Scientific (Gibco™). Cells were grown under humidified 5% CO_2 in air at 37°C and were regularly tested for mycoplasma contamination. To enhance the UC emission for lifetime imaging, a sample of DPA@ureasil containing a 16x increase in sensitiser concentration (12.8 µM) was used.

Chinese Hamster Ovary (CHO) cells were initially purchased from ECACC (UK). Cells were grown in phenol red free DMEM/F-12 (Dulbecco's modified Eagle Medium/Nutrient Mixture F-12) supplemented with 10% FBS, and 1% Penicillin-Streptomycin. Cells were seeded at a density of 2.5 x 10^5 cells/well in 8 well or 32 mm glass bottom chamber slides (Ibidi). 24 h after cell seeding, aliquots of DPA@ureasil solution (200 µL, with 3 mg HEPES (2-[4-(2-hydroxyethyl)piperazin-1-yl]ethanesulfonic acid) added before use to lower pH) were added to the dishes so that a dilution of 100 was achieved. The cells were allowed to uptake the NPs for at least 2 h before imaging. Control samples that were co-seeded with the nucleic acid stain Hoechst 33342 were also prepared to investigated localization of the NPs in the cell structure.

Confocal images were taken using an inverted Nikon Ti-E microscope attached to a Nikon EC2 scanning unit. The confocal scanning unit was equipped with a Super K Extreme NKT-SC 470-2000 nm supercontinuum laser (NKT Photonics) (79 MHz repetition rate; 70 ps pulse

width). Imaging was acquired with a 60x (NA 1.27) water immersion objective. Microscopy images of luminescence (long-lived) emission were detected through a 500 nm short pass filter (Thorlabs FESH0500, and a 460 ± 60 nm). The excitation wavelengths were 532 nm, selected using an AOTF (NKT super K select) for the NPs, and 405 nm for the Hoechst 33342 counterstain. Although short-lived (< 10ns) TREM ("FLIM") data may be acquired using a modified Nikon EC2 confocal[8], while longer-lived (>100 ns) ("PLIM") data could be acquired by reducing the laser repetition rate to kHz, this is generally an inefficient method for long-lived TREM measurements as the number of photons detected is too low for accurate lifetime calculation. Instead, the long-lived TREM images from the NP UC emission were acquired using a Becker & Hickl DCS120 confocal unit coupled to a time-correlated single photon (TCSPC) SPC150 module equipped with a HPM100-40 detector running the TCSPC software v 9.77. The high repetition rate laser (79 MHz) was used to continuously excite the sample. Emission photons detected were characterized by their time in the laser pulse period and the coordinates of the laser spot in the scanning area in the moment of its detection in a standard TCSPC method, whilst the x,y coordinates from a scanning system generate the lifetime image. The DCS120 scanning system (pixel clock and x,y galvanometers signal) is importantly synchronously modulated (on/off cycles) in time with the high repetition NKT laser. In this configuration, FLIM can be recorded during the 'On' time, and 'PLIM' or any long-lived emission during the 'Off' time. The SPCM software then generates separate images for the short and the long-lived emission which are then analysed with SPCImage analysis software. We note that TREM measurements were performed using an open pinhole, while steady-state imaging was performed using a medium pinhole, leading to better resolution.

3 Supporting Experimental Data

3.1 Dynamic Light Scattering (DLS)

DLS measurements were performed using a Zetasizer Nano® (Nano-ZS, Malvern Instruments, UK) equipped with a He-Ne laser at 633 nm with a 4 mW power source. The measurement was performed in backscatter mode at an angle of 173°. The hydrodynamic diameter (D_h) was calculated by assuming that the diffusing particles were monodisperse spheres. The D_h was averaged from 3 runs of approximately 15 scans each. Cumulants analysis, which is a fit of the logarithm of the correlation function, gave a mean value for the size (Z-average) and dispersity index. Prior to measurement, the DPA@ureasil suspension was diluted 5-fold by water and filtered with a 0.2 µm nylon syringe filter.

a

b

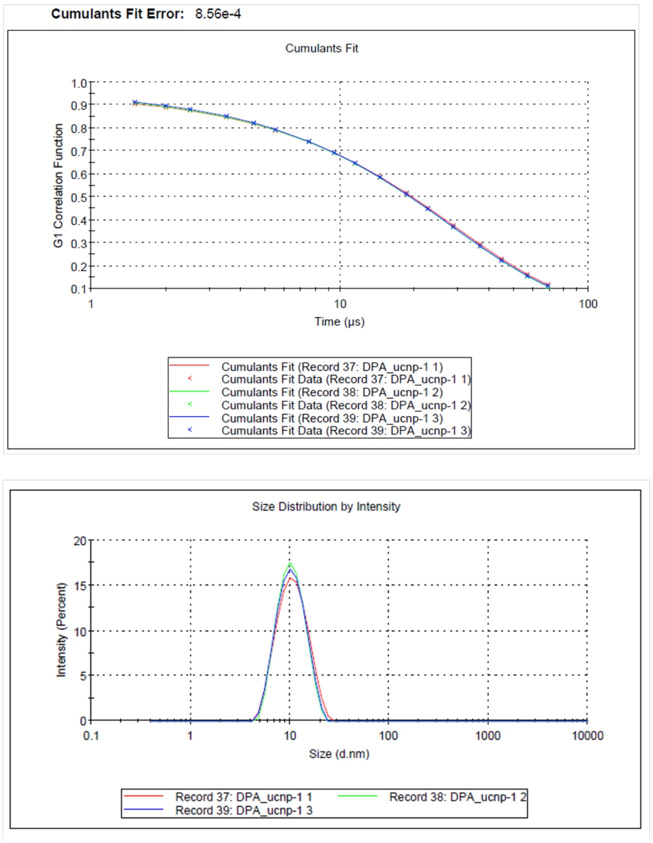

Figure S1 | a. DLS correlogram with cumulants fit and **b**. The extracted size distribution by intensity for DPA@ureasil. The sample was measured three times.

3.2 Effect of Oxygen on Upconversion Emission for TTA@Ureasils

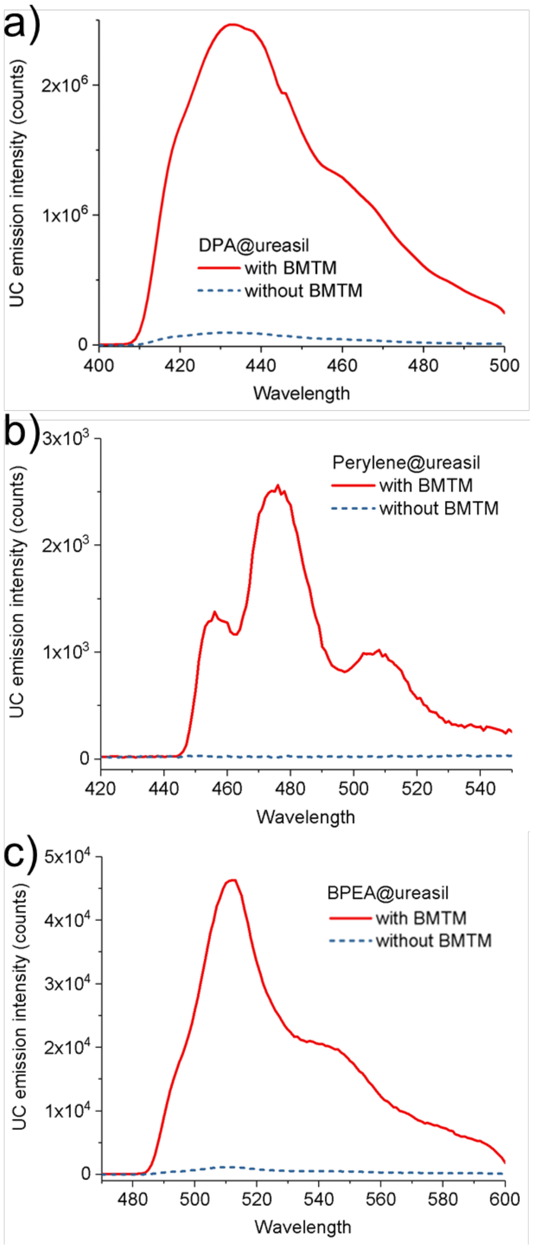

Figure S2 I UC emission spectra of **a.** DPA@ureasil (λ_{exc} = 532 nm, 2000 mW cm^{-2}), **b.** Perylene@ureasil and **c.** BPEA@ureasil (both λ_{exc} = 635 nm, 7.5 mW cm^{-2}) and their counterpart samples with no oxygen scavenger (BMTM).

3.3 The TTET rate of DPA@ureasil.

As shown in Figure S3, the phosphorescence of PtOEP in DPA@ureasil is only partially quenched by the emitter compared to a control sample of PtOEP@ureasil (no DPA emitter). The quantum yield of TTET (Φ_{TTET}) for DPA@ureasil was determined from:[9]

$$\Phi_{TTET} = 1 - \frac{I_{phos}}{I_{0,phos}} \qquad \text{(Eq.S1)}$$

where I_{phos} and $I_{0,phos}$ are the integrated intensity of the phosphorescence band of the DPA@ureasil and the sensitiser-only PtOEP@ureasil sample, respectively, measured under identical conditions. The calculated Φ_{TTET} of 52% for DPA@ureasil is comparable to similar TTA-UC NP systems reported.[9,10] We note that Φ_{TTET} in NPs is typically much lower than observed in solution phase TTA-UC systems (close to unity)[11] as the process of NP assembly is kinetically controlled and leads to a non-uniform distribution of luminophores.[10,12] As such there will be a population of NPs with a lower than average sensitiser concentration, which decreases the rate of TTET. Moreover, the elevated emitter concentration means that DPA may form aggregates within the NPs, resulting in local phase separation with the sensitiser, which further decreases Φ_{TTET}.

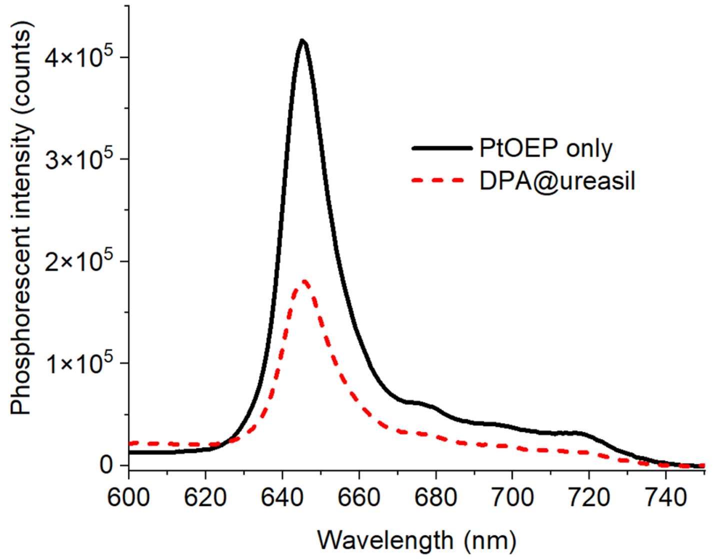

Figure S3 I Phosphorescence spectra of DPA@ureasil (red dashed line) and PtOEP@ureasil (no DPA emitter, black line), measured under 532 nm laser excitation (500 mW cm^{-2} with a long-pass filter at 550 nm).

3.4 UC emission lifetime decay studies

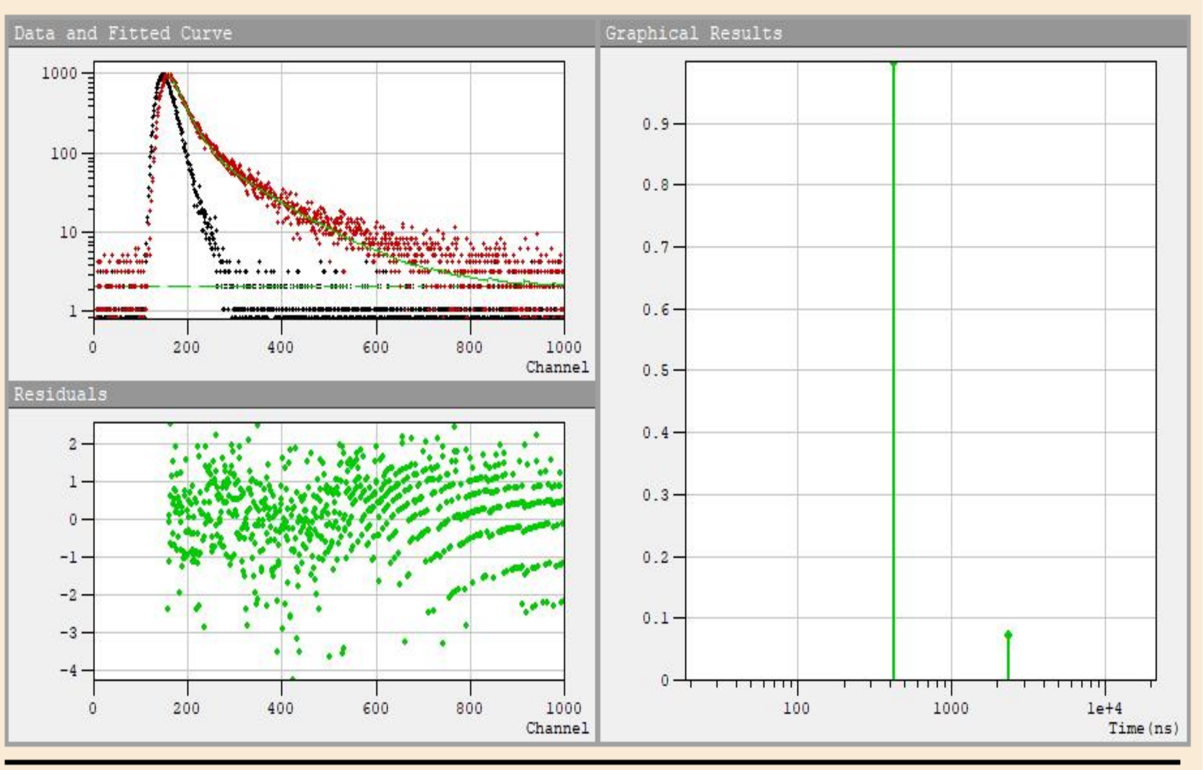

File: decay_20us_1kc&IRF.FL

❖ **Exponential Components Analysis (Reconvolution)**

Fitting range : [155; 1000] channels

χ^2 : 1.144

	B_i	f_i	τ_i (ns)
1	0.0729	71.486	406.888
2	0.0052	28.514	2279.572

Shift : -300.00 ns

Decay Background : 2.000 fixed

IRF background : 0.700

Figure S4 I Quality and extracted parameters to a biexponential fit to the UC emission decay of DPA@ureasil (λ_{exc} = 532 nm, λ_{em} = 440 nm) in FAST (Edinburgh Instruments). The residuals (green) ad chi-squared (χ^2) indicate a good fit to the data.

3.5 Cell imaging studies

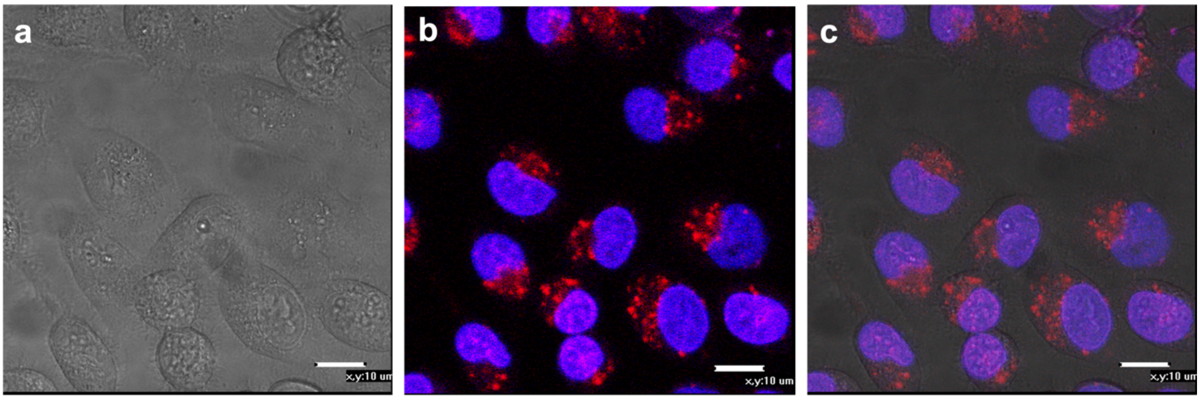

Figure S5 I Grey scale **a.** and emission **b.** images of CHO cells co-labelled with Hoechst 33342 and DPA@ureasil after 24 hours. In (b) selective excitation (exc) and emission (em) wavelengths enable isolation of emission from Hoechst 33342 (blue, λ_{exc} = 405 nm, λ_{em} = = 460 nm) and the NPs (red PtOEP emission, λ_{exc} = 488 nm, λ_{em} = 605 nm), respectively. **c.** is an overlay image of (a) and (b). Scale bar = 10 µm. Hoechst 33342 is a selective nuclear stain, while the DPA@ureasil NPs (red) predominantly localize outside of the nucleus, albeit with some permeation. Notably, after 24 hours, the cells look healthy (flat), with no signs of membrane blebbing or debris in the medium, suggesting that the NPs exhibit low cytotoxicity. We note that the DPA@ureasil NPs aggregated after 24 hours, observed as the red particles in the above figures, potentially due to metabolic degradation of the ureasil host.

3.6 Other TTA-UC@ureasil systems

To demonstrate the universal compatibility of ureasil NPs, two different TTA-UC luminophore pairs were incorporated, namely PdTPBP/perylene (perylene@ureasil) and PdTPBP/BPEA (BPEA@ureasil). Aqueous suspensions of perylene@ureasil and BPEA@ureasil are transparent, but highly coloured due to the strong absorbance of the emitters (Figures S6a, b). Both the absorption and emission spectra of dilute perylene@ureasil and BPEA@ureasil were slightly red-shifted in comparison with the analogous emitter solution in THF, suggesting that there may be some aggregation of the emitters in both NP systems (Figures S6c,d). Beyond this, the optical properties remained otherwise unchanged.

UC emission was observed from both NP suspensions upon laser excitation at 635 nm (Figures S6a, b). Similar to DPA@ureasil, the UC emission spectra of perylene@ureasil and BPEA@ureasil showed strong reabsorption due to the high emitter concentration. Addition of the O_2 scavenger similarly enhanced the UC emission intensity (~80 and 42 times, respectively, for perylene@ureasil and BPEA@ureasil, Figures S2b, S2c) compared to the BMTM-free analogues. Overall, the photophysical trends for perylene@ureasil and BPEA@ureasil suspensions were very similar to that of DPA@ureasil, suggesting that the

ureasil NP system should be compatible with a wide variety of other TTA-UC luminophore pairs.

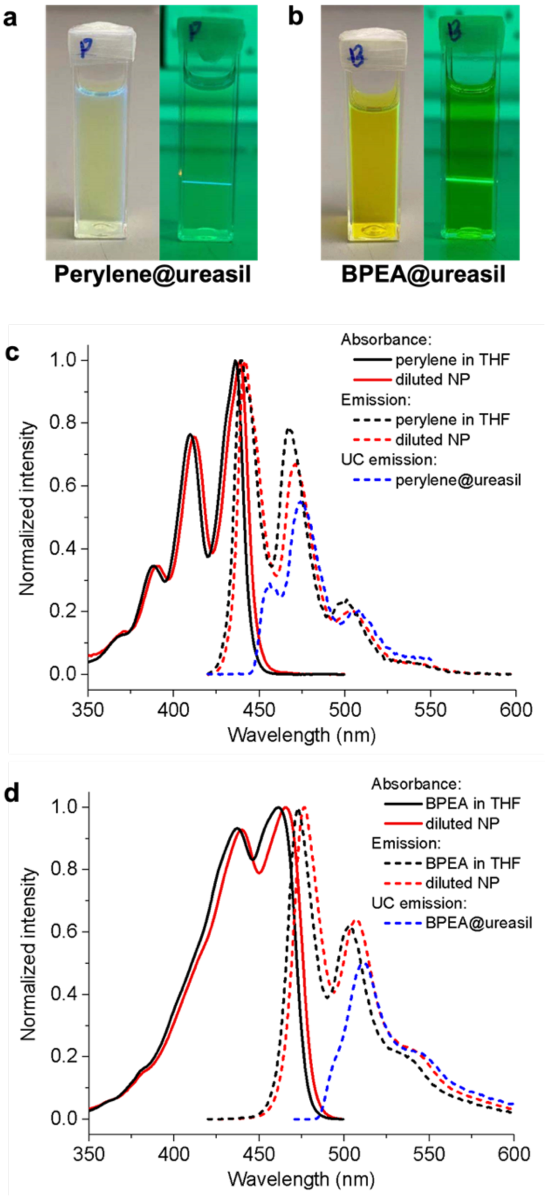

Figure S6 | Optical properties of other TTA@ureasil NPs. **a, b.** Photographs of Perylene@ureasil and BPEA@ureasil, respectively, under ambient light and 635 nm laser excitation (600 nm short-pass filter). Normalized absorption and emission spectra of **c.** Perylene in THF (1×10^{-5} M) and perylene@ureasil and **d.** BPEA in THF (1×10^{-5} M) and BPEA@ureasil. Diluted NP samples were prepared by diluting the original NP suspension 100-fold with water. Both PL emission spectra were taken at λ_{exc} = 400 nm. The UC emission spectra of the original NP suspensions (λ_{exc} = 635 nm, short-pass filter cut-off at 600 nm) were plotted by tail normalisation to the PL emission spectrum of diluted NP suspension.

4 References

(1) Sader, K.; Matadeen, R.; Castro Hartmann, P.; Halsan, T.; Schlichten, C. Industrial Cryo-EM Facility Setup and Management. *Acta Crystallogr. Sect. D Struct. Biol.* **2020**, *76* (4), 313–325. https://doi.org/10.1107/S2059798320002223.

(2) Zivanov, J.; Nakane, T.; Forsberg, B. O.; Kimanius, D.; Hagen, W. J.; Lindahl, E.; Scheres, S. H. New Tools for Automated High-Resolution Cryo-EM Structure Determination in RELION-3. *Elife* **2018**, *7*. https://doi.org/10.7554/eLife.42166.

(3) Rohou, A.; Grigorieff, N. CTFFIND4: Fast and Accurate Defocus Estimation from Electron Micrographs. *J. Struct. Biol.* **2015**, *192* (2), 216–221. https://doi.org/10.1016/j.jsb.2015.08.008.

(4) Grant, T.; Rohou, A.; Grigorieff, N. CisTEM, User-Friendly Software for Single-Particle Image Processing. *Elife* **2018**, *7*. https://doi.org/10.7554/eLife.35383.

(5) Berg, S.; Kutra, D.; Kroeger, T.; Straehle, C. N.; Kausler, B. X.; Haubold, C.; Schiegg, M.; Ales, J.; Beier, T.; Rudy, M.; Eren, K.; Cervantes, J. I.; Xu, B.; Beuttenmueller, F.; Wolny, A.; Zhang, C.; Koethe, U.; Hamprecht, F. A.; Kreshuk, A. Ilastik: Interactive Machine Learning for (Bio)Image Analysis. *Nat. Methods* **2019**, *16* (12), 1226–1232. https://doi.org/10.1038/s41592-019-0582-9.

(6) Schneider, C. A.; Rasband, W. S.; Eliceiri, K. W. NIH Image to ImageJ: 25 Years of Image Analysis. *Nat. Methods* **2012**, *9* (7), 671–675. https://doi.org/10.1038/nmeth.2089.

(7) Brouwer, A. M. Standards for Photoluminescence Quantum Yield Measurements in Solution (IUPAC Technical Report). *Pure Appl. Chem.* **2011**, *83* (12), 2213–2228. https://doi.org/10.1351/PAC-REP-10-09-31.

(8) Botchway, S. W.; Scherer, K. M.; Hook, S.; Stubbs, C. D.; Weston, E.; Bisby, R. H.; Parker, A. W. A Series of Flexible Design Adaptations to the Nikon E-C1 and E-C2 Confocal Microscope Systems for UV, Multiphoton and FLIM Imaging. *J. Microsc.* **2015**, *258* (1), 68–78. https://doi.org/10.1111/jmi.12218.

(9) Huang, L.; Le, T.; Huang, K.; Han, G. Enzymatic Enhancing of Triplet–Triplet Annihilation Upconversion by Breaking Oxygen Quenching for Background-Free Biological Sensing. *Nat. Commun.* **2021**, *12* (1), 1898. https://doi.org/10.1038/s41467-021-22282-1.

(10) Mattiello, S.; Monguzzi, A.; Pedrini, J.; Sassi, M.; Villa, C.; Torrente, Y.; Marotta, R.; Meinardi, F.; Beverina, L. Self-Assembled Dual Dye-Doped Nanosized Micelles for High-Contrast Up-Conversion Bioimaging. *Adv. Funct. Mater.* **2016**, *26* (46), 8447–8454. https://doi.org/10.1002/adfm.201603303.

(11) Gao, C.; Prasad, S. K. K.; Zhang, B.; Dvořák, M.; Tayebjee, M. J. Y.; McCamey, D. R.; Schmidt, T. W.; Smith, T. A.; Wong, W. W. H. Intramolecular Versus Intermolecular Triplet Fusion in Multichromophoric Photochemical Upconversion. *J. Phys. Chem. C* **2019**, *123* (33), 20181–20187. https://doi.org/10.1021/acs.jpcc.9b07098.

(12) Gao, C.; Zhang, B.; Hall, C. R.; Li, L.; Chen, Y.; Zeng, Y.; Smith, T. A.; Wong, W. W. H. Triplet Fusion Upconversion Using Sterically Protected 9,10-Diphenylanthracene as the Emitter. *Phys. Chem. Chem. Phys.* **2020**, *22* (11), 6300–6307. https://doi.org/10.1039/c9cp06311k.
